# Crystal structures of the μ2 subunit of clathrin-adaptor protein 2 in complex with peptides derived from human papillomavirus 16 E7

**DOI:** 10.71150/jm.2505003

**Published:** 2025-08-31

**Authors:** Sujin Jung, Dahwan Lim, Joon Sig Choi, Ho-Chul Shin, Seung Jun Kim, Bonsu Ku

**Affiliations:** 1 Orphan Disease Therapeutic Target Research Center, Korea Research Institute of Bioscience and Biotechnology, Daejeon 34141, Republic of Korea; 2 Department of Biochemistry, Chungnam National University, Daejeon 34134, Republic of Korea; 3 Critical Diseases Diagnostics Convergence Research Center, Korea Research Institute of Bioscience and Biotechnology, Daejeon 34141, Republic of Korea

**Keywords:** clathrin-adaptor protein 2, AP2, μ2, human papillomavirus, HPV16, E7, CR2, crystal structure

## Abstract

Human papillomaviruses (HPVs) cause abnormal cellular proliferation, leading to malignant or benign lesions, such as cervical cancer and warts. The genome of HPV16, the most prevalent high-risk oncogenic genotype within the *Alphapapillomavirus* genus, encodes two oncoproteins. One of these proteins, E7, interacts with multiple host proteins and modulates their functions through distinct pathways. The CR2 domain of HPV16 E7 was recently reported to interact with the μ2 subunit of clathrin-adaptor protein 2 (AP2-μ2), an adaptor complex involved in cargo internalization during clathrin-mediated endocytosis. In this study, to provide molecular insights into their intermolecular interactions, we determined the crystal structures of AP2-μ2 in complex with the HPV16 E7-derived peptides. Subsequent biochemical analyses revealed that this interaction is primarily maintained by the Y-x-x-Φ motif and further supported by acidic cluster residues of HPV16 E7. Finally, sequence alignment of the E7 CR2 domains from various HPV genotypes showed that the AP2-μ2-binding motif is largely conserved in *Alpha*-, *Beta*-, and *Mupapillomaviruses*, but not in *Nu*- and *Gammapapillomaviruses*.

## Introduction

Human papillomaviruses (HPVs) are non-enveloped, double-stranded DNA viruses that infect mucosal or cutaneous tissues, causing cellular hyperproliferation and leading to a variety of benign or malignant lesions (Harden and Munger, 2017; Tommasino, 2014). HPVs belong to the *Papillomaviridae* family, which comprises more than 200 genotypes classified into five genera: *Alpha*-, *Beta*-, *Mu*-, *Nu*-, and *Gammapapillomavirus* (Egawa et al., 2015). Among these, HPV16, 18, 31, 33, 35, 39, 45, 51, 52, 56, 58, 59, 66, and 68, belonging to the *Alphapapillomavirus* genus, are considered high-risk genotypes, as these are major oncoviruses responsible for approximately 98% of cervical cancers and 7% of head and neck cancers (Serrano et al., 2018). Especially, HPV16 is the most prevalent high-risk genotype, responsible for approximately 60% of HPV-associated cervical cancers (LeConte et al., 2018). The remaining HPV genotypes are considered low-risk, and their role in tumorigenesis remains unclear (Bandolin et al., 2020; Tahseen et al., 2021).

The HPV genome generally encodes seven functional “Early” and two capsid-forming “Late” proteins. Among them, E6 and E7 are the two viral oncoproteins directly implicated in tumorigenesis upon HPV infection (Mittal and Banks, 2017). Numerous molecular and structural studies have revealed diverse interactions between host proteins and HPV E6/E7, providing a foundation for understanding the role and mechanisms of these two viral oncoproteins (Han et al., 2024; Harden and Munger, 2017; Lee et al., 1998, 2021; Martinez-Zapien et al., 2016; Mittal and Banks, 2017; Yun et al., 2019). Recently, a novel host protein that interacts with E7 of HPV16 was identified: the μ2 subunit of the clathrin-adaptor protein 2 (AP2) complex (Basukala et al., 2022).

AP2 is a complex consisting of four subunits (α, β2, μ2, and σ2) that localizes to the plasma membrane, where it serves as an adaptor for cargo internalization during clathrin-mediated endocytosis (Beacham et al., 2019; Mettlen et al., 2018). The μ2 subunit of AP2, referred to as AP2-μ2 in this study, mediates the selective recruitment of diverse cargo proteins into nascent clathrin-coated vesicles by recognizing and directly binding to the Y-x-x-Φ motif. These cargoes include epidermal growth factor receptor (EGFR) (Owen and Evans, 1998; Sorkin and Goh, 2009), type I phosphatidylinositol-4-phosphate 5-kinase isoform γ (PIP5KI_γ_) (Kahlfeldt et al., 2010; Rohde et al., 2002), and integrin α_4_ (De Franceschi et al., 2016). Numerous viral proteins containing the Y-x-x-Φ motif also interact with AP2-μ2 to exploit this complex for viral replication, assembly, entry, trafficking, or release (Yuan et al., 2020). Notable examples include Gag and Vpu of human immunodeficiency virus type 1 (Batonick et al., 2005; Stoneham et al., 2017), core protein of hepatitis C virus (Neveu et al., 2012), and glycoprotein B of pseudorabies virus (Van Minnebruggen et al., 2004).

In 2022, Basukala et al. (2022) reported a de novo association between AP2-μ2 and HPV16 E7. This interaction, which depends on the Y^25^-E-Q-L^28^ sequence within the conserved region 2 (CR2) domain of E7, facilitates cell transformation and sustains EGFR endocytosis. They also suggested that Ser31 and Ser32 in the CR2 domain of HPV16 E7, located immediately after the Y^25^-E-Q-L^28^ motif and phosphorylated by casein kinase II (CKII), are involved in the interaction with AP2-μ2, as alanine substitutions of these residues noticeably impaired complex formation (Basukala et al., 2022). In this study, we characterized these associations in detail by determining the crystal structures of AP2-μ2 bound to three HPV16 E7-derived peptides and by performing biochemical analyses of the binding between recombinant AP2-μ2 and various synthetic peptides derived from HPV E7. We also analyzed interactions between AP2-μ2 and the E7 CR2 domains from diverse HPV genotypes, demonstrating that this interaction is broadly, but not entirely, conserved across the five HPV genera.

## Materials and Methods

### Preparation, crystallization, and structural determination

A DNA fragment encoding residues 158–435 of the μ2 subunit of rat AP2 was cloned into a modified pET28a plasmid (Novagen, USA) to produce a recombinant protein tagged with (His)_10_−maltose-binding protein. The recombinant protein was expressed in *Escherichia coli* BL21(DE3) RIL cells (Novagen) grown in Luria-Bertani medium at 18°C for 16 h following induction with 0.5 mM isopropyl β-D-thiogalactopyranoside. Proteins were purified using Ni-NTA affinity chromatography (QIAGEN, Germany), followed by size-exclusion chromatography on a HiLoad 26/600 Superdex 200 prep grade column (Cytiva, USA). The (His)_10_−maltose-binding protein tag was cleaved by TEV protease and removed during subsequent purification steps. Final protein samples were equilibrated in a buffer containing 50 mM Tris-HCl (pH 7.5), 500 mM NaCl, and 3 mM β-mercaptoethanol. Purified AP2-μ2 was mixed with each of the HPV16 E7(22–32; S31E∙S32E), HPV16 E7(22–39; S31E∙S32E), and HPV16 E7(22–39) synthetic peptides at a 1:3 molar ratio and incubated for 15 h. Crystals were obtained using the sitting-drop vapor diffusion method at 18°C by from a mixture of 1 μl protein solution (10 mg/ml) and 1 μl precipitant solution. The crystallization conditions were 2.5 M sodium chloride and 100 mM potassium phosphate monobasic/sodium phosphate dibasic (pH 6.2) for the HPV16 E7(22–32; S31E∙S32E)-bound form, 3.5 M ammonium chloride and 100 mM sodium acetate trihydrate (pH 4.5) for the HPV16 E7(22–39; S31E∙S32E)-bound form, and 2.0 M sodium formate and 100 mM sodium acetate trihydrate (pH 4.6) for the HPV16 E7(22–39)-bound form. X-ray diffraction data were collected at beamlines 5C and 7A of the Pohang Accelerator Laboratory (Korea) and processed using *HKL2000* (Otwinowski and Minor, 1997). Molecular replacement was performed using Phaser (McCoy et al., 2007) with the structure of AP2-μ2 complexed with a PIP5KI_γ_-derived peptide (Protein Data Bank [PDB] code: 3H85) used as the search model (Kahlfeldt et al., 2010). Model building and refinement were performed using *Coot* (Emsley and Cowtan, 2004) and *PHENIX* (Adams et al., 2010), respectively. All crystallographic data are presented in Table 1.

### Sample preparation for isothermal titration calorimetry measurements

Recombinant AP2-μ2 protein harboring R402A∙K405A mutations was prepared and purified using the same protocol as that used for the wild-type protein. Synthetic peptides derived from HPV16 E7, including 11-mer (residues 22–32; wild-type, Y25H, Y25N, L28A, or S31E∙S32E), 17-mer (residues 16–32), and 18-mer (residues 22–39; wild-type or S31E∙S32E), as well as 18-mer peptides derived from HPV4, HPV15, and HPV18 E7 (residues 24–41, 24–41, and 25–42, respectively), were purchased from Dandicure (Korea). All peptides were dialyzed against the same buffer used for AP2-μ2 purification. Isothermal titration calorimetry (ITC) was performed using recombinant AP2-μ2 proteins and synthetic peptides as previously described (Jung et al., 2023; Lim et al., 2021).

## Results

### Crystal structures of AP2-μ2 complexed with HPV16 E7 CR2 peptide

**Fig. 1.**
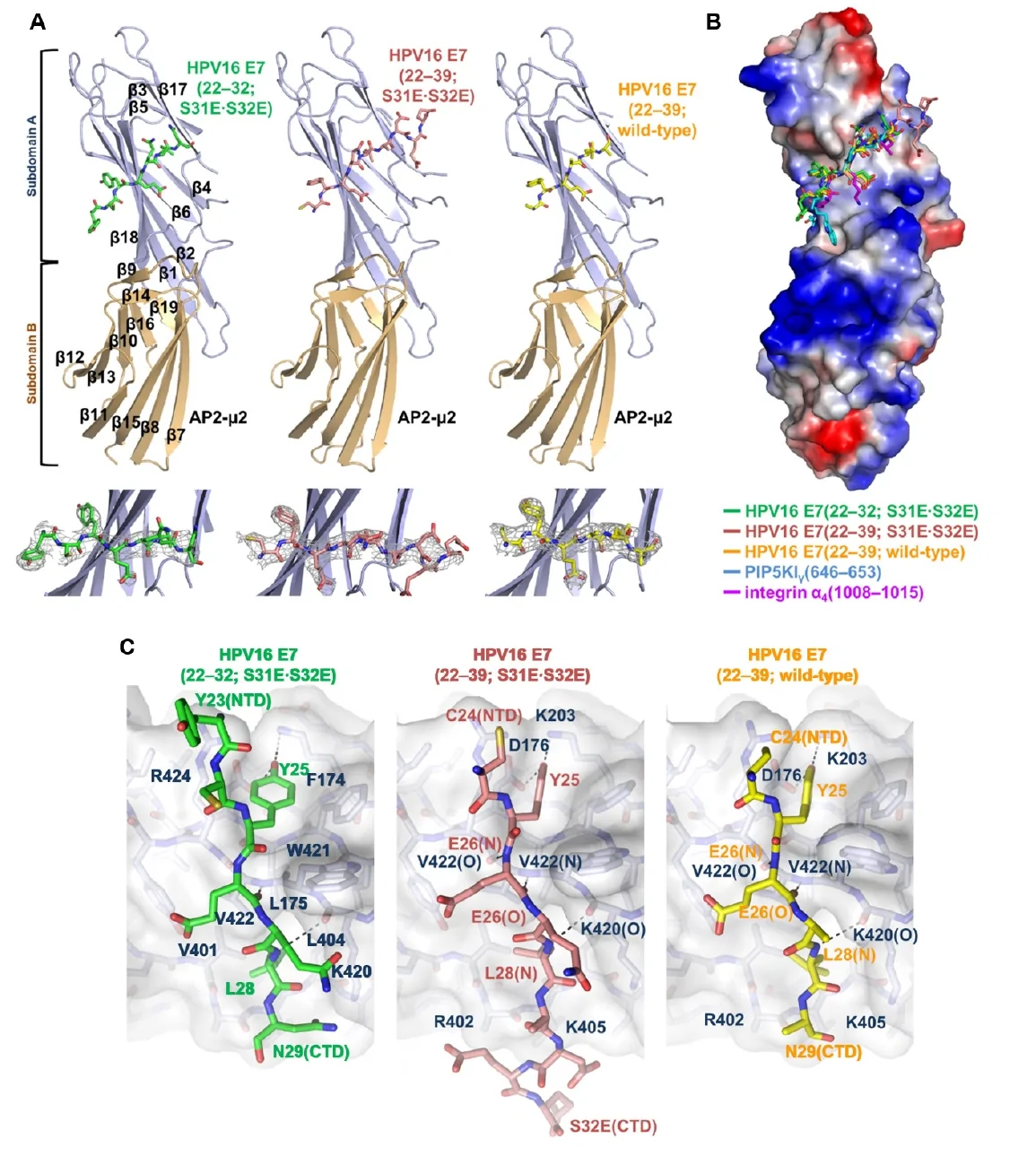
Structural analysis of three AP2-μ2−HPV16 E7-derived peptide complexes. (A) Overall structural representation. (Top) AP2-μ2 is shown as ribbon diagrams, and HPV16 E7-derived peptides are depicted as sticks. Secondary structure elements of AP2-μ2 are labeled only in the HPV16 E7(22–32; S31E∙S32E)-bound structure for clarity. (Bottom) HPV16 E7-derived peptides are shown as sticks, overlaid with 2m*F*_o_-D*F*_c_ electron density omit maps contoured at 1.0 σ. (B) Five AP2-μ2-bound peptides are shown as sticks and aligned. Electrostatic surface representation is provided only for AP2-μ2 bound to the HPV16 E7(22–32; S31E∙S32E) peptide. PDB codes are 3H85 (AP2-μ2-bound PIP5KI_γ_) and 5FPI (AP2-μ2-bound integrin α_4_). (C) Atomic details of the interactions between AP2-μ2 and the three HPV16 E7-derived peptides. Residues at the amino and carboxyl termini, as well as those primarily involved in binding, are labeled. For clarity, residues mediating intermolecular hydrophobic interaction are labeled at left, whereas those participating in hydrogen bonding or electrostatic interactions are labeled in the center and at right. Dashed lines indicate hydrogen bonds.

To investigate the intermolecular interaction between AP2-μ2 and the CR2 domain of HPV16 E7 at atomic resolution, we determined the crystal structures of AP2-μ2 in complex with three CR2 domain-derived peptides using X-ray crystallography. For crystallization, recombinant AP2-μ2 was incubated with each of the three CR2 domain-derived peptides at a 1:3 molar ratio: HPV16 E7(22–32; S31E∙S32E), HPV16 E7(22–39; wild-type), and HPV16 E7(22–39; S31E∙S32E). The S31E∙S32E substitution was introduced at two peptides to mimic the CKII-dependent phosphorylated form. Crystals belonging to the space group *P*6_4_ were obtained and used for structure determination of the three complexes (Table 1). In all three structures, AP2-μ2 consists of two β-sandwich-folded subdomains: subdomain A (β1–β6 and β17–β18) and subdomain B (β7–β16 and β19) (Fig. 1A). The three HPV16 E7-bound AP2-μ2 structures superimposed well with each other and with the apo AP2-μ2 structure (PDB code: 7OFP), with root mean square deviation values of 0.19–0.44 Å over 212–249 aligned C_α_ atoms. These results indicate that binding to any of the three HPV16 E7 peptides did not significantly alter the conformation of AP2-μ2. Subdomain A of AP2-μ2 contains a central ligand-binding pocket that accommodates the HPV16 E7 peptides in a canonical manner, similar to previously determined crystal structures of AP2-μ2 bound to PIP5KI_γ_- or integrin α_4_-derived peptides (De Franceschi et al., 2016; Kahlfeldt et al., 2010) (Fig. 1B).

Next, we analyzed the intermolecular interaction between AP2-μ2 and HPV16 E7 in detail. In all three structures, Tyr25 and Leu28 in the Y-x-x-Φ motif of HPV16 E7 play critical roles in complex formation by mediating hydrophobic interactions with Phe174, Leu175, Val401, Leu404, Lys420, Trp421, Val422, and Arg424 of AP2-μ2 (Fig. 1C). The side chain hydroxyl group of Tyr25 in HPV16 E7 further contributes to binding by forming hydrogen bonds with the side chain carboxyl group of Asp176 and amino group of Lys203 in AP2-μ2 (Fig. 1C). The AP2-μ2−HPV16 E7 interaction is further stabilized by multiple hydrogen bonds involving main chain atoms: the amide nitrogen atoms of Glu26 and Leu28 in HPV16 E7 and Val422 in AP2-μ2 and the carbonyl oxygen atoms of Glu26 in HPV16 E7 and Lys420 and Val422 in AP2-μ2 (Fig. 1C).

### Y-x-x-Φ motif of HPV16 E7 is critical for binding to AP2-μ2

**Fig. 2.**
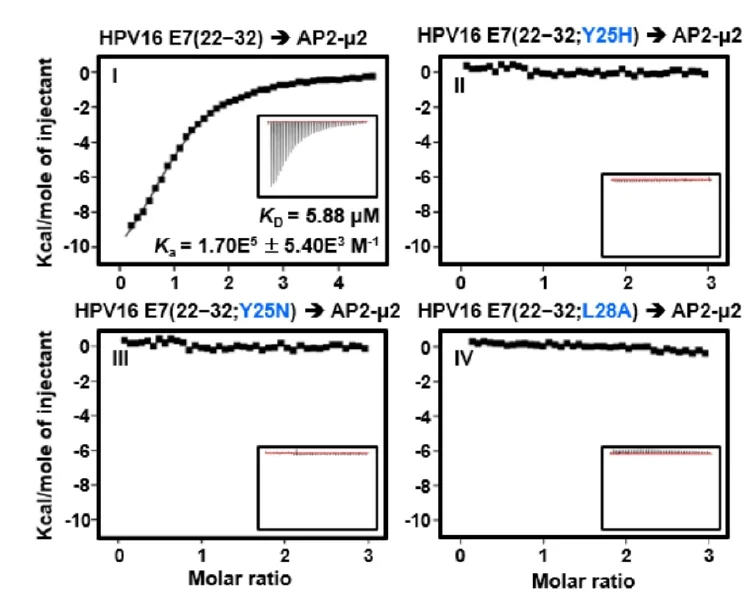
ITC analysis of the effect of the Y-x-x-Φ motif on complex formation. Each peptide (0.2 mM) was titrated into 10 μM recombinant AP2-μ2 as indicated. *K*_D_ and *K*_a_ values were deduced from curve fitting of the integrated heat per mole of added ligand. Mutated residues are highlighted in blue.

To quantitatively assess the interaction between AP2-μ2 and HPV16 E7, recombinant AP2-μ2 protein (residues 158–435) expressed in *E. coli* and a panel of synthetic HPV16 E7-derived peptides were prepared, and their binding affinities were measured using ITC. First, the 11-mer wild-type peptide comprising residues 22–32 of HPV16 E7 (L^22^-Y-C-Y-E-Q-L-N-D-S-S^32^) bound directly to AP2-μ2 with a dissociation constant (*K*_D_) of 5.88 μM (Fig. 2, panel I). This affinity was similar to that of an 8-mer Y-x-x-Φ motif-containing peptide derived from PIP5KI_γ_ (S^646^-W-V-Y-S-P-L-H^653^; *K*_D_ of 6 μM), and substantially stronger than that of the 8-mer peptide derived from integrin α_4_ (Q^1008^-Y-K-S-I-L-Q-E^1015^; *K*_D_ of 72 μM), which were measured using the same method (De Franceschi et al., 2016; Kahlfeldt et al., 2010). Disruption of the Y-x-x-Φ motif through mutations (Y25H, Y25N, or L28A) severely impaired the binding interaction between AP2-μ2 and the HPV16 E7(22–32) peptide (Fig. 2, panels II–IV), demonstrating the essential role of this motif in complex formation.

### Acidic cluster region of HPV16 E7 contributes to the interaction with AP2-μ2

**Fig. 3.**
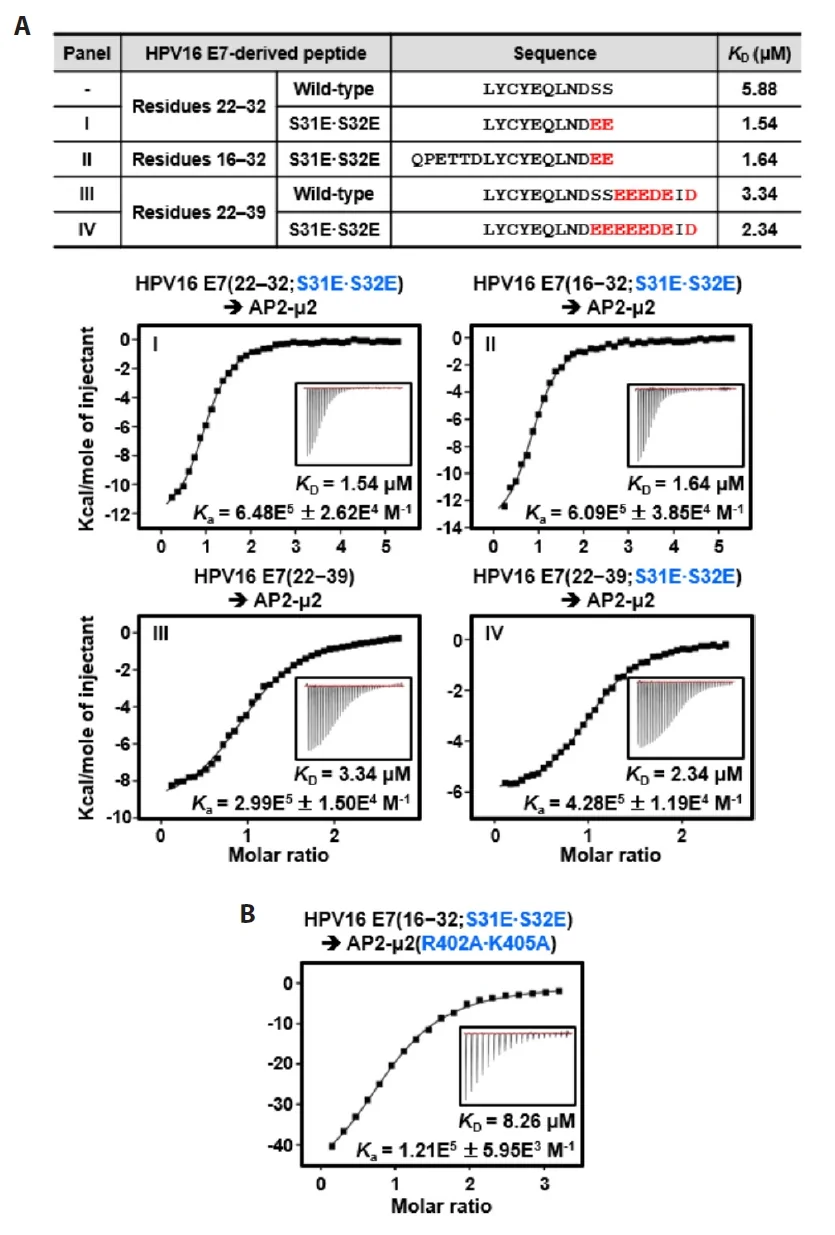
ITC analysis of the effect of the acidic cluster region on complex formation. Each peptide was titrated into wild-type (A) or R402A∙K405A mutant (B) recombinant AP2-μ2 as indicated. *K*_D_ and *K*_a_ values were obtained by curve-fitting of the integrated heat per mole of added ligand. Mutated residues are highlighted in blue. The peptides analyzed in this experiment, along with their amino acid sequences and AP2-μ2-binding affinities, are listed in the table shown at the top. Negatively charged cluster residues are marked in red.

We next analyzed the contribution of the acidic cluster region of HPV16 E7, which lies downstream of the Y^25^-E-Q-L^28^ sequence and contains multiple negatively charged residues, to binding to AP2-μ2. An 11-mer phosphomimetic mutant HPV16 E7 peptide, in which the two serine residues were substituted with glutamate (L^22^-Y-C-Y-E-Q-L-N-D-E-E^32^), was synthesized and subjected to ITC analysis. The *K*_D_ of the mutant peptide, HPV16 E7(22–32; S31E∙S32E), was 1.54 μM (Fig. 3A, panel I), which was nearly four-fold higher than that of the wild-type HPV16 E7(22–32) peptide (*K*_D_ of 5.88 μM; Fig. 2, panel I). These results suggest that substituting the two glutamate residues for the tandem serine motif (Ser31 and Ser32) of HPV16 E7 substantially enhanced the binding interaction with AP2-μ2. They are consistent with previous biochemical data showing that CKII-dependent phosphorylation of the CR2 domain of HPV16 E7 enhances its binding to AP2-μ2 (Basukala et al., 2022). Extending the N-terminus of the mutant peptide by six residues had a minimal effect on its binding affinity to AP2-μ2 (*K*_D_ of 1.64 μM), indicating that residues 16–22 of HPV16 E7 are not critical for the interaction (Fig. 3A, panel II). We also prepared two C-terminally extended HPV16 E7 peptides (residues 22–39), which included seven additional amino acids (E^33^-E-E-D-E-I-D^39^). The binding affinities of HPV16 E7(22–39) and HPV16 E7(22–39; S31E∙S32E) to AP2-μ2 were 3.34 and 2.34 μM, respectively (Fig. 3A, panels III and IV), further supporting that phosphorylation of the two serine residues contributes positively to the complex formation. We also found that HPV16 E7(22–39) exhibited a higher affinity to AP2-μ2 (*K*_D_ of 3.34 μM; Fig. 3A, panel III) compared to HPV16 E7(22–32) (*K*_D_ of 5.88 μM; Fig. 2, panel I), suggesting that negatively charged residues downstream of the Y^25^-E-Q-L^28^ sequence in HPV16 E7, such as Glu33, Glu34, Glu35, Asp36, Glu37, and Asp39, facilitate binding to AP2-μ2. However, the S31E∙S32E substitution and negatively charged residues at positions 33–39 did not synergistically enhance AP2-μ2−HPV16 E7 complex formation, as the HPV16 E7(22–39; S31E∙S32E) peptide exhibited lower binding affinity to AP2-μ2 (*K*_D_ of 2.34 μM; Fig. 3A, panel IV) than the HPV16 E7(22–32; S31E∙S32E) peptide (*K*_D_ of 1.54 μM; Fig. 3A, panel I). Therefore, phosphorylation of Ser31 and Ser32 in HPV16 E7 by CKII may support Y^25^-E-Q-L^28^ motif-dependent complex formation with AP2-μ2, presumably by mediating electrostatic interactions with positively charged residues of AP2-μ2, such as Arg402 and Lys405. However, even in the unphosphorylated state, the four glutamate and two aspartate residues downstream of the Y^25^-E-Q-L^28^ motif in HPV16 E7 CR2 appeared to support its binding to AP2-μ2, possibly by also engaging with positively charged residues of AP2-μ2. Given the lack of synergistic enhancement in binding to AP2-μ2, the S31E∙S32E substitution and negatively charged residues at positions 33–39 of HPV16 E7 likely compete for interaction with the same positively charged residues of AP2-μ2.

Despite the contribution of the acidic cluster region to complex formation demonstrated in biochemical analyses (Figs. 2 and 3), most acidic cluster residues of the CR2 domain of HPV16 E7 were not resolved in any of the three crystal structures (Fig. 1). For instance, in the crystal structure of AP2-μ2−HPV16 E7(22–32; S31E∙S32E) complex, only residues 23–29 (Y^23^-C-Y-E-Q-L-N^29^) were visible, whereas Leu22, Asp30, and the phosphomimetic substitutions Ser31Glu and Ser32Glu were not (Fig. 1C, left). Weak electron density also prevented the tracing of residues 22–23 (Leu22 and Tyr23) and 30–39 (D^30^-S-S-E-E-E-D-E-I-D^39^) during refinement of the AP2-μ2−HPV16 E7(22–39; wild-type) complex structure (Fig. 1C, right). Similarly, in the crystal structure of AP2-μ2 bound to the HPV16 E7(22–39; S31E∙S32E) peptide, only residues 24–32 (C^24^-Y-E-Q-L-N-D-S31E-S32E^32^) were visible in the electron density map after refinement (Fig. 1C, middle). Moreover, the side chains of Arg402 and Lys405 in AP2-μ2, the two positively charged residues thought to interact with the negatively charged residues of HPV16 E7, were not visible in any of the three complex crystal structures (Fig. 1C). Therefore, the acidic cluster residues within the CR2 domain of HPV16 E7 may contribute to complex formation by modulating loosely packed electrostatic attractions among multiple negatively charged residues from HPV16 E7 and positively charged residues of AP2-μ2 rather than forming specific one-to-one residue-level interactions readily detectable by crystallographic analysis. Consistently, alanine substitution of Arg402 and Lys405 in AP2-μ2, previously suggested to mediate interactions with negatively charged residues of HPV16 E7 (Basukala et al., 2022), showed reduced binding affinity to the HPV16 E7(16–32; S31E∙S32E) peptide by approximately five-fold (*K*_D_ of 8.26 μM; Fig. 3B). This finding further supports that transient and non-specific electrostatic contacts between AP2-μ2 and the CR2 domain of HPV16 E7 are involved in complex stabilization.

### AP2-μ2-binding motif in E7 is conserved across multiple, but not all, HPV genotypes

**Fig. 4.**
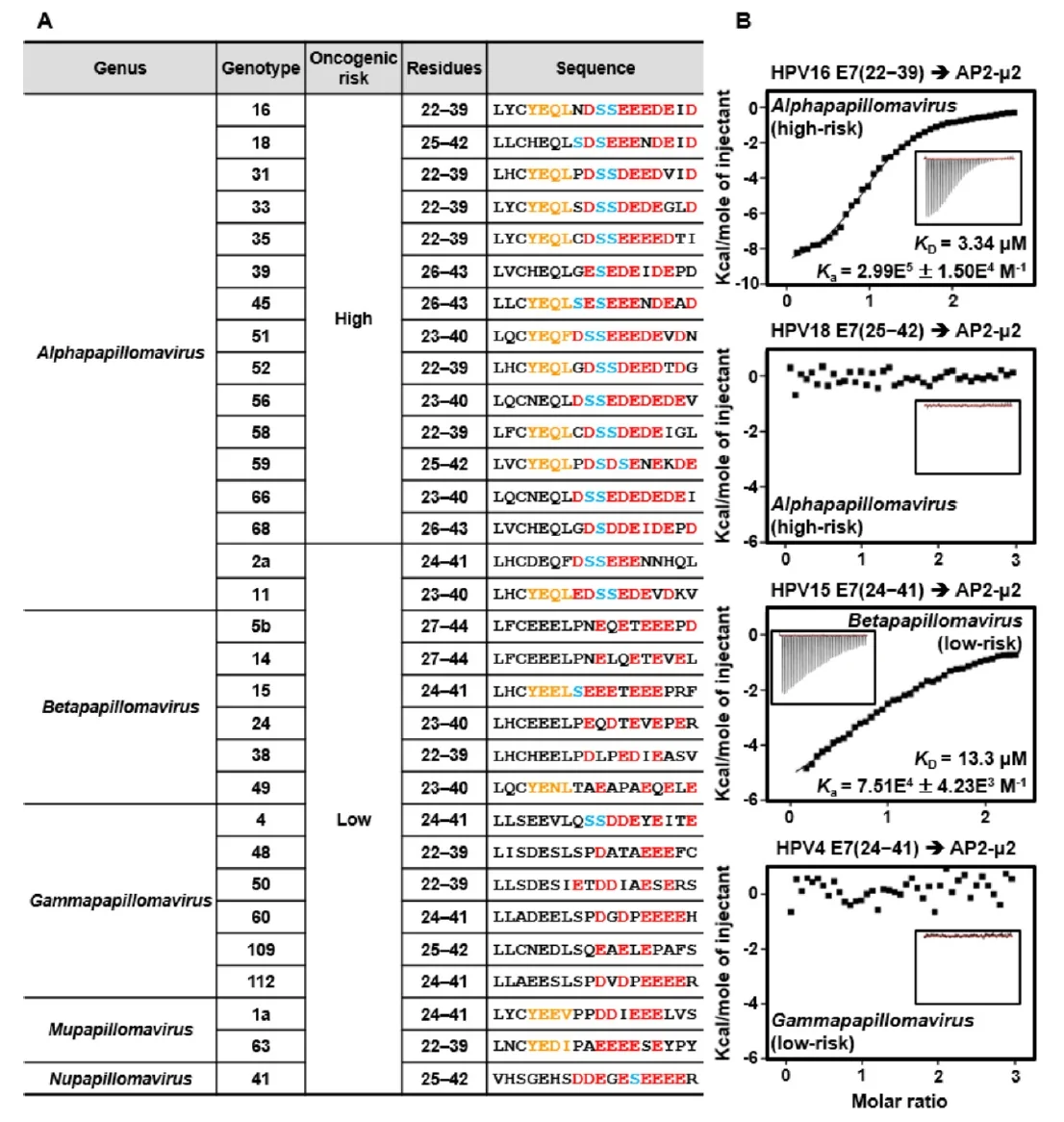
Sequence alignment of the CR2 domain of E7 among various HPV genotypes. (A) Sequence alignment of the CR2 domains from the E7 proteins of 31 representative HPV genotypes. The Y-x-x-Φ motif, CKII phosphorylation motif (S-x-x-D/E), and the cluster of negatively charged residues following the Y-x-x-Φ motif are highlighted in orange, light blue, and red, respectively. (B) AP2-μ2-binding affinities of 18-mer peptides derived from the E7 proteins of HPV16 (shown in Fig. 3A, panel III), HPV18, HPV15, and HPV4 were measured using ITC.

We next analyzed whether the AP2-μ2-binding motifs in the CR2 domain of HPV16 E7 are conserved among other HPV genotypes. Amino acid sequences corresponding to residues 22–39 of HPV16 E7 were aligned across 14 high-risk and two representative low-risk *Alphapapillomaviruses*, 12 representative *Beta*- and *Gammapapillomaviruses*, and three defined *Mu*- and *Nupapillomaviruses* (Fig. 4A). The Y-x-x-Φ motif, which plays a critical role in binding of HPV16 E7 to AP2-μ2 (Figs. 1 and 2), is conserved in 9 of the 14 high-risk *Alphapapillomaviruses*, as well as in several low-risk *Alpha*-, *Beta*-, and *Mupapillomaviruses*, but is absent in *Gamma*- and *Nupapillomaviruses* (Fig. 4A). Serine residues corresponding to the consensus CKII-dependent phosphorylation motif (S-x-x-E/D) were identified in most *Alphapapillomavirus* E7 proteins, such as Ser31 and Ser32 in HPV16 E7 and Ser 32 and Ser34 in HPV18 E7, but were rare in E7 proteins from other genera (Fig. 4A). By contrast, the acidic cluster that is thought to play a supportive rather than an essential role in binding to AP2-μ2 (Fig. 3) was conserved across all papillomaviruses analyzed in this study (Fig. 4A). In ITC experiments, peptides lacking the Y-x-x-Φ motif comprising residues 25–42 of HPV18 E7 (a high-risk *Alphapapillomavirus*) or residues 24–41 of HPV4 E7 (a *Gammapapillomavirus*) did not bind to AP2-μ2, demonstrating that the acidic cluster alone is insufficient to sustain the interaction (Fig. 4B). Contrastingly, the Y-x-x-Φ motif-containing peptide derived from HPV15 E7 (a *Betapapillomavirus*) bound AP2-μ2 with a *K*_D_ of 13.3 μM (Fig. 4B). Collectively, the AP2-μ2-binding region, characterized by the presence of the Y-x-x-Φ motif and acidic cluster residues, is found in most, but not all, high-risk *Alphapapillomavirus* E7 proteins. Thus, AP2-μ2−HPV E7 binding-dependent inhibition of EGFR internalization, which contributes to cellular transformation, may be a strategy employed not only by HPV16 but also by several other oncogenic HPV genotypes. Some low-risk papillomaviruses may also target AP2-μ2 during infection, as our analysis revealed that the Y-x-x-Φ motif followed by an acidic cluster is present in the CR2 domain of E7 proteins from several low-risk *Alpha*-, *Beta*-, and *Mupapillomaviruses*.

## Discussion

In this study, we employed a combination of structural (Fig. 1) and biochemical (Figs. 2–4) approaches to investigate the role and significance of the Y^25^-E-Q-L^28^ motif and acidic cluster region within the CR2 domain of HPV16 E7 in binding to AP2-μ2. Our results demonstrate that the Y^25^-E-Q-L^28^ motif of HPV16 E7 is essential for complex formation with AP2-μ2 (Figs. 1 and 2). Notably, this sequence partially overlaps with the Rb1-binding motif (L^22^-Y-C-Y-E^26^) of HPV16 E7. Therefore, the CR2 domain of HPV16 E7 can recognize not only Rb1, a major tumor suppressor and well-known E7-binding host protein, but also AP2-μ2, a key adaptor protein involved in cargo internalization during clathrin-mediated endocytosis, presumably in a competitive manner. The subcellular localization of HPV16 E7 may be a key factor determining its binding preference between the two proteins, as Rb1 is primarily localized in the nucleus (Zhu, 2005), whereas AP2-μ2 is tethered to the plasma membrane (Beacham et al., 2019; Mettlen et al., 2018).

Our data indicate that negatively charged residues downstream of the Y^25^-E-Q-L^28^ motif, including two phosphomimetic glutamate substitutions for serines (Ser31Glu and Ser32Glu), four glutamates (Glu33, Glu34, Glu35, and Glu37), and two aspartates (Asp36 and Asp39), also contribute to the interaction with AP2-μ2 (Fig. 3). However, unlike the Y^25^-E-Q-L^28^ motif, the acidic cluster region of HPV16 E7 appears to play a supportive rather than a primary role in binding to AP2-μ2. This interpretation is supported by the crystal structures, in which most acidic cluster residues were not resolved (Fig. 1), and by the observation that the acidic cluster-deficient HPV16 E7(22–32) peptide retained considerable binding affinity for AP2-μ2 (*K*_D_ of 5.88 μM; Fig. 2A). Additionally, the phosphomimetic mutations only modestly increased binding affinity (Fig. 3A). Therefore, the acidic cluster region may serve as a secondary contributor to AP2-μ2 binding, in addition to its previously reported roles, such as stabilizing the extended conformation of E7 (Kukic et al., 2019) and promoting efficient S-phase entry of infected cells (Firzlaff et al., 1991; Genovese et al., 2008).

Nevertheless, we do not exclude the possibility that the use of E7 peptides containing phosphomimetic substitutions may have limited conformational stabilization, thus hindering the stable association with AP2-μ2. This notion is supported by a previous study, which reported that phosphorylation, rather than glutamate substitution, was necessary to fully recapitulate the conformational stabilization and binding interactions (Ha et al., 2004). Additionally, the resolution limits of our data, which ranged from 3.2–3.7 Å (Table 1), may have affected the visualization of acidic cluster residues, which were not clearly resolved in the crystal structures (Fig. 1). Therefore, to precisely determine the role of phosphorylation, HPV16 E7 peptides containing phosphoserines at positions Ser31 and Ser32 should be prepared to analyze their interactions with AP2-μ2 either by determining the complex structure at higher resolution or by measuring their binding affinity. This will be the focus of future investigation.

The *Papillomaviridae* family includes more than 200 HPV genotypes, whose oncoproteins vary not only in amino acid sequences but also in their interacting partners. For example, E6 proteins from high-risk genotypes contain the PDZ domain-binding motif at the C-terminal end, whereas those from low-risk HPV genotypes lack this feature (Lee et al., 2022). In addition, HPVs classified within the *Gammapapillomavirus* genus recognize Rb1 through their CR3 domain, because of the absence of the canonical Rb1-binding L-x-C-x-E motif in their CR2 domain (Wang et al., 2010). The two most common oncogenic HPVs, HPV16 and HPV18, contain an N-terminal L-x-C-x-E motif that mediates recruitment of Rb1. However, although the C-terminal CR3 domain of HPV16 contributes to Rb1 binding, the corresponding domain in HPV18 does not, despite that both viruses belong to the *Alphapapillomavirus* genus (Todorovic et al., 2012). Similarly, we found that the Y-x-x-Φ motif in E7, which is critical for AP2-μ2 binding, is not conserved across all HPV genotypes (Fig. 4). Thus, the presence or absence of the AP2-μ2-binding Y-x-x-Φ motif in the CR2 domain of HPV E7 represents another layer of sequence variation distinguishing different HPV genotypes. The underlying reason for the conservation of this motif in certain HPV genotypes requires further analysis.

### Concluding Remark

We performed structural and biochemical analyses of the complex formed between the μ2 subunit of AP2 and the CR2 domain of HPV16 E7. Our findings provide a molecular basis for understanding the multiple functions of the HPV E7 oncoprotein, which not only promotes the degradation of tumor suppressor proteins, but also modulates the internalization of various host proteins such as EGFR. These oncoprotein-dependent viral activities likely act in concert to promote cellular transformation during HPV infection.

## Figures and Tables

**Table 1.** Data collection and structure refinement statistics

Protein	AP2-μ2−HPV16 E7 (22–32; S31E∙S32E)	AP2-μ2−HPV16 E7 (22–39; S31E∙S32E)	AP2-μ2−HPV16 E7 (22–39)
**PDB code**	9UUK	9UUL	9UUJ
**Data Collection**			
Space group	*P*6_4_	*P*6_4_	*P*6_4_
Unit cell dimensions			
a, b, c (Å)	125.2, 125.2, 73.5	125.6, 125.6, 74.3	125.1, 125.1, 73.6
α, β, γ (^o^)	90, 90, 120	90, 90, 120	90, 90, 120
Resolution (Å)	50.0−3.2 (3.26−3.20)^a^	50.0−3.3 (3.36−3.30)	50.0−3.3 (3.76−3.70)
*R*_sym_^b^ (%)	6.3 (36.7)	4.3 (38.8)	7.4 (48.2)
*I*/σ (*I*)	17.5 (1.6)	15.7 (1.6)	21.0 (3.2)
Completeness (%)	97.4 (97.0)	97.8 (90.7)	94.7 (96.9)
Redundancy	5.1	5.1	7.3
**Refinement**			
Resolution (Å)	50.0−3.2	50.0−3.3	50.0−3.7
Number of reflections	10714	9960	6751
*R*_work_^c^/*R*_free_ (%)	20.5/24.4	19.4/22.5	20.7/23.4
Number of atoms			
Protein	1876	1933	1944
Peptide	64	75	45
RMSD			
Bond lengths (Å)	0.011	0.011	0.003
Bond angles (^o^)	1.213	1.131	0.618
Ramachandran plot (%)			
Most favored region	92.3	92.8	93.5
Additionally allowed region	7.7	7.2	6.5
Average B-values (Å^2^)			
Protein	87.8	96.0	112.6
Peptide	104.2	120.2	123.8

a The numbers in parentheses are statistics from the shell with the highest resolution.

b *R*_sym_ = Σ |*I*_obs_ - *I*_avg_| / *I*_obs_, where *I*_obs_ is the observed intensity of individual reflection and *I*_avg_ is the average across symmetry equivalents.

c *R*_work_ = Σ ||*F*_o_| - |*F*_c_|| / Σ |*F*_o_|, where |*F*_o_| and |*F*_c_| are the observed and calculated structure factor amplitudes, respectively. *R*_free_ was calculated with 9.9–10.1% of the data.

## Data Availability

The coordinates of the structures, together with the structure factors, were deposited in PDB under accession code 9UUK, 9UUL, and 9UUJ for AP2-μ2−HPV16 E7(22–32; S31E∙S32E), AP2-μ2−HPV16 E7(22–39; S31E∙S32E), and AP2-μ2−HPV16 E7(22–39), respectively.

## References

[b1-jm-2505003] Adams PD, Afonine PV, Bunkóczi G, Chen VB, Davis IW (2010). *PHENIX*: a comprehensive Python-based system for macromolecular structure solution. Acta Crystallogr D Biol Crystallogr.

[b2-jm-2505003] Bandolin L, Borsetto D, Fussey J, Da Mosto MC, Nicolai P (2020). *Beta* human papillomaviruses infection and skin carcinogenesis. Rev Med Virol.

[b3-jm-2505003] Basukala O, Trejo-Cerro O, Myers MP, Pim D, Massimi P (2022). HPV-16 E7 interacts with the endocytic machinery via the AP2 adaptor μ2 subunit. mBio.

[b4-jm-2505003] Batonick M, Favre M, Boge M, Spearman P, Höning S (2005). Interaction of HIV-1 Gag with the clathrin-associated adaptor AP-2. Virology.

[b5-jm-2505003] Beacham GM, Partlow EA, Hollopeter G (2019). Conformational regulation of AP1 and AP2 clathrin adaptor complexes. Traffic.

[b6-jm-2505003] De Franceschi N, Arjonen A, Elkhatib N, Denessiouk K, Wrobel AG (2016). Selective integrin endocytosis is driven by interactions between the integrin α-chain and AP2. Nat Struct Mol Biol.

[b7-jm-2505003] Egawa N, Egawa K, Griffin H, Doorbar J (2015). Human papillomaviruses; epithelial tropisms, and the development of neoplasia. Viruses.

[b8-jm-2505003] Emsley P, Cowtan K (2004). Coot: model-building tools for molecular graphics. Acta Crystallogr D Biol Crystallogr.

[b9-jm-2505003] Firzlaff JM, Luscher B, Eisenman RN (1991). Negative charge at the casein kinase II phosphorylation site is important for transformation but not for Rb protein binding by the E7 protein of human papillomavirus type 16. Proc Natl Acad Sci USA.

[b10-jm-2505003] Genovese NJ, Banerjee NS, Broker TR, Chow LT (2008). Casein kinase II motif-dependent phosphorylation of human papillomavirus E7 protein promotes p130 degradation and S-phase induction in differentiated human keratinocytes. J Virol.

[b11-jm-2505003] Ha NC, Tonozuka T, Stamos JL, Choi HJ, Weis WI (2004). Mechanism of phosphorylation-dependent binding of APC to β-catenin and its role in β-catenin degradation. Mol Cell.

[b12-jm-2505003] Han F, Guo XY, Jiang MX, Xia NS, Gu Y (2024). Structural biology of the human papillomavirus. Structure.

[b13-jm-2505003] Harden ME, Munger K (2017). Human papillomavirus molecular biology. Mutat Res Rev Mutat Res.

[b14-jm-2505003] Jung S, Lee HS, Shin HC, Choi JS, Kim SJ (2023). Crystal structures of Plk1 polo-box domain bound to the human papillomavirus minor capsid protein L2-derived peptide. J Microbiol.

[b15-jm-2505003] Kahlfeldt N, Vahedi-Faridi A, Koo SJ, Schäfer JG, Krainer G (2010). Molecular basis for association of PIPKIγ-p90 with clathrin adaptor AP-2. J Biol Chem.

[b16-jm-2505003] Kukic P, Lo Piccolo GM, Nogueira MO, Svergun DI, Vendruscolo M (2019). The free energy landscape of the oncogene protein E7 of human papillomavirus type 16 reveals a complex interplay between ordered and disordered regions. Sci Rep.

[b17-jm-2505003] LeConte BA, Szaniszlo P, Fennewald SM, Lou DI, Qiu S (2018). Differences in the viral genome between HPV-positive cervical and oropharyngeal cancer. PLoS One.

[b18-jm-2505003] Lee HS, Kim MW, Jin KS, Shin HC, Kim WK (2021). Molecular analysis of the interaction between human PTPN21 and the oncoprotein E7 from human papillomavirus genotype 18. Mol Cells.

[b19-jm-2505003] Lee JO, Russo AA, Pavletich NP (1998). Structure of the retinoblastoma tumour-suppressor pocket domain bound to a peptide from HPV E7. Nature.

[b20-jm-2505003] Lee HS, Yun HY, Lee EW, Shin HC, Kim SJ (2022). Structural and biochemical analysis of the PTPN4 PDZ domain bound to the C-terminal tail of the human papillomavirus E6 oncoprotein. J Microbiol.

[b21-jm-2505003] Lim D, Shin HC, Choi JS, Kim SJ, Ku B (2021). Crystal structure of human LC8 bound to a peptide from Ebola virus VP35. J Microbiol.

[b22-jm-2505003] Martinez-Zapien D, Ruiz FX, Poirson J, Mitschler A, Ramirez J (2016). Structure of the E6/E6AP/p53 complex required for HPV-mediated degradation of p53. Nature.

[b23-jm-2505003] McCoy AJ, Grosse-Kunstleve RW, Adams PD, Winn MD, Storoni LC (2007). Phaser crystallographic software. J Appl Crystallogr.

[b24-jm-2505003] Mettlen M, Chen PH, Srinivasan S, Danuser G, Schmid SL (2018). Regulation of clathrin-mediated endocytosis. Annu Rev Biochem.

[b25-jm-2505003] Mittal S, Banks L (2017). Molecular mechanisms underlying human papillomavirus E6 and E7 oncoprotein-induced cell transformation. Mutat Res Rev Mutat Res.

[b26-jm-2505003] Neveu G, Barouch-Bentov R, Ziv-Av A, Gerber D, Jacob Y (2012). Identification and targeting of an interaction between a tyrosine motif within hepatitis C virus core protein and AP2M1 essential for viral assembly. PLoS Pathog.

[b27-jm-2505003] Otwinowski Z, Minor W (1997). Processing of X-ray diffraction data collected in oscillation mode. Methods Enzymol.

[b28-jm-2505003] Owen DJ, Evans PR (1998). A structural explanation for the recognition of tyrosine-based endocytotic signals. Science.

[b29-jm-2505003] Rohde G, Wenzel D, Haucke V (2002). A phosphatidylinositol (4,5)-bisphosphate binding site within μ2-adaptin regulates clathrin-mediated endocytosis. J Cell Biol.

[b30-jm-2505003] Serrano B, Brotons M, Bosch FX, Bruni L (2018). Epidemiology and burden of HPV-related disease. Best Pract Res Clin Obstet Gynaecol.

[b31-jm-2505003] Sorkin A, Goh LK (2009). Endocytosis and intracellular trafficking of ErbBs. Exp Cell Res.

[b32-jm-2505003] Stoneham CA, Singh R, Jia X, Xiong Y, Guatelli J (2017). Endocytic activity of HIV-1 Vpu: Phosphoserine-dependent interactions with clathrin adaptors. Traffic.

[b33-jm-2505003] Tahseen D, Rady PL, Tyring SK (2021). Effects of β-HPV on DNA damage response pathways to drive carcinogenesis: a review. Virus Genes.

[b34-jm-2505003] Todorovic B, Hung K, Massimi P, Avvakumov N, Dick FA (2012). Conserved region 3 of human papillomavirus 16 E7 contributes to deregulation of the retinoblastoma tumor suppressor. J Virol.

[b35-jm-2505003] Tommasino M (2014). The human papillomavirus family and its role in carcinogenesis. Semin Cancer Biol.

[b36-jm-2505003] Van Minnebruggen G, Favoreel HW, Nauwynck HJ (2004). Internalization of pseudorabies virus glycoprotein B is mediated by an interaction between the YQRL motif in its cytoplasmic domain and the clathrin-associated AP-2 adaptor complex. J Virol.

[b37-jm-2505003] Wang J, Zhou D, Prabhu A, Schlegel R, Yuan H (2010). The canine papillomavirus and gamma HPV E7 proteins use an alternative domain to bind and destabilize the retinoblastoma protein. PLoS Pathog.

[b38-jm-2505003] Yuan S, Chu H, Huang J, Zhao X, Ye ZW (2020). Viruses harness YxxØ motif to interact with host AP2M1 for replication: a vulnerable broad-spectrum antiviral target. Sci Adv.

[b39-jm-2505003] Yun HY, Kim MW, Lee HS, Kim W, Shin JH (2019). Structural basis for recognition of the tumor suppressor protein PTPN14 by the oncoprotein E7 of human papillomavirus. PLoS Biol.

[b40-jm-2505003] Zhu L (2005). Tumour suppressor retinoblastoma protein Rb: a transcriptional regulator. Eur J Cancer.

